# Perceiving fingers in single-digit arithmetic problems

**DOI:** 10.3389/fpsyg.2015.00226

**Published:** 2015-03-16

**Authors:** Ilaria Berteletti, James R. Booth

**Affiliations:** ^1^Department of Communication Sciences and Disorders, Northwestern University, Evanston, IL, USA; ^2^Department of Psychology, University of Illinois at Urbana–Champaign, Champaign, IL, USA; ^3^Department of Communication Sciences and Disorders, The University of Texas at Austin, Austin, TX, USA

**Keywords:** finger gnosia, arithmetic facts, somatosensory, motor, arithmetic skill

## Abstract

In this study, we investigate in children the neural underpinnings of finger representation and finger movement involved in single-digit arithmetic problems. Evidence suggests that finger representation and finger-based strategies play an important role in learning and understanding arithmetic. Because different operations rely on different networks, we compared activation for subtraction and multiplication problems in independently localized finger somatosensory and motor areas and tested whether activation was related to skill. Brain activations from children between 8 and 13 years of age revealed that only subtraction problems significantly activated finger motor areas, suggesting reliance on finger-based strategies. In addition, larger subtraction problems yielded greater somatosensory activation than smaller problems, suggesting a greater reliance on finger representation for larger numerical values. Interestingly, better performance in subtraction problems was associated with lower activation in the finger somatosensory area. Our results support the importance of fine-grained finger representation in arithmetical skill and are the first neurological evidence for a functional role of the somatosensory finger area in proficient arithmetical problem solving, in particular for those problems requiring quantity manipulation. From an educational perspective, these results encourage investigating whether different finger-based strategies facilitate arithmetical understanding and encourage educational practices aiming at integrating finger representation and finger-based strategies as a tool for instilling stronger numerical sense.

## Introduction

Historically and among different cultures, humans have been relying on fingers or body parts to support their representation of numbers (Ifrah, 1994). In occidental cultures, counting on fingers is one of the first strategies taught to children to link the verbal representation of a number with its numerical meaning (Gelman and Gallistel, 1978; Gallistel and Gelman, 1992; Butterworth, 1999; Sato and Lalain, 2008). When counting and calculation procedures are not automatized, fingers alleviate working memory and are important visual cues (Barrouillet and Lépine, 2005; Barrouillet et al., 2008). Children with mathematical learning difficulties rely more on fingers compared to peers either to help represent numerical quantities or to facilitate the execution of operation-specific procedures (Alibali and DiRusso, 1999; Geary, 2005). To date, little is known about the role of finger-based strategies in shaping and creating a strong numerical and arithmetical understanding. Indeed, greater understanding of the neurofunctional changes induced by the use of different finger strategies and their relation to skill will allow implementing better educational practices and hopefully provide alternative tools to remediate mathematical learning difficulties.

Hand and finger representations have been shown to influence children and adults at different levels of numerical processing (Di Luca et al., 2006; Di Luca and Pesenti, 2008; Domahs et al., 2008; Badets and Pesenti, 2010; Badets et al., 2010). Early external finger-based configurations, used to represent numbers and support calculation procedures, become internalized during primary school to the point of influencing adult performance when performing numerical tasks (Di Luca et al., 2006; Di Luca and Pesenti, 2008; Klein et al., 2011). Prototypical finger configurations in adults were responded to faster than atypical ones in an Arabic digit-to-finger mapping task and only these gave an automatic access to number semantics (Di Luca et al., 2006; Di Luca and Pesenti, 2008). Finger-based strategies also influence mental arithmetic (Badets et al., 2010; Klein et al., 2011; Newman and Soylu, 2013). For instance, adults like children show an effect in mental calculation resulting from a failure in keeping track of “full hands” known as the split-five error (i.e., answers to the problems deviated exactly by ±5 from the correct result; Domahs et al., 2008; Klein et al., 2011). In addition, internalized finger-based representation of arithmetic operations may still rely on motor and somatosensory finger areas in adults since passive finger movement was found to disrupt counting-based strategies during simple mental addition problems (Imbo et al., 2011). Importantly, not only the motor component of finger-based strategies and the mental representation of hand configurations are related to numerical and arithmetical processing, but evidence shows that finger representation *per se*, known as finger gnosia, is related to skill. A lesion in the dominant inferior parietal lobule is found to cause Gerstmann’s syndrome (Gerstmann, 1940) where finger agnosia (i.e., deficit in finger representation) and acalculia (i.e., disability to calculate) are associated suggesting that both competences rely, at least partially, on common processes. The relation between finger gnosia and arithmetic skill has also been found in children independently from IQ scores (Strauss and Werner, 1938; Costa et al., 2011). In 6-year-olds, the quality of the finger representation was a better predictor of mathematical skill than standard developmental tests (Fayol et al., 1998; Noël, 2005; Reeve and Humberstone, 2011) and training in finger discrimination at the same age improved performance to numerical and quantification tasks (Gracia-Bafalluy and Noël, 2008). Interestingly, the development of finger gnosis, non-symbolic numerical abilities and spatial abilities have been found to be correlated independently from age thus further supporting a functional link between the different competences (Chinello et al., 2013).

Both electrophysiological and neuroimaging studies have found evidence for common neural substrates for hand representation and numerical processing. Using transcranial magnetic stimulation (TMS), Sato et al. (2007) reported changes of excitability of hand muscles in participants performing a visual parity judgment task. Similarly, Andres et al. (2007) found changes of excitability in a visual counting task irrespective of the nature of the counting sequence (i.e., numbers or letters) suggesting that hand motor circuits may assist the counting process by keeping track of one-to-one correspondence. In an fMRI study, Kansaku et al. (2007) showed that the left ventral premotor cortex was specifically activated when counting large sequences. In the same study, the crucial role of this area for counting large sequences (over 20 elements) was confirmed by applying TMS, which disrupted participants’ counting ability. Only one fMRI study independently localized the hand motor representation to investigate number processing related activation (Tschentscher et al., 2012). Despite the absence of overt hand movements, perceiving numbers as digits or written words enhanced activation in motor and premotor areas contralateral to the preferred hand for counting (i.e., left vs. right hand starters).

Activations in finger-related areas have also been reported during calculation. The left precentral finger area, often combined with fronto-lateral activations, has been found active in several studies (Dehaene, 1996; Rueckert et al., 1996; Pesenti et al., 2000; Stanescu-Cosson et al., 2000; Zago et al., 2001) suggesting that these activations reflect the involvement of a finger-movement network underlying finger counting (Pesenti et al., 2000). The premotor and frontal cortices were also found to be significantly more activated during single-digit additions compared to verbal rehearsal (Hanakawa et al., 2002), were specifically activated for two-digit addition and subtraction problems jointly with bilateral parietal areas (Knops and Willmes, 2014), and were sensitive to difficulty in single-digit multiplication problems (Jost et al., 2009). In a meta-analysis, Arsalidou and Taylor (2011) showed that across fMRI studies, the prefrontal cortex including the precentral gyrus is significantly active in all four basic arithmetic tasks. Indeed, the strong co-occurrence of activation in prefrontal cortex (MFG and premotor cortex) and the IPS has also been highlighted in a recent review on the neurobiological underpinning of mathematical cognition (Ashkenazi et al., 2013).

A strong relation is found between finger representation and arithmetic skill, and neuroimaging studies indicate finger-related activations during calculations but to our knowledge only two studies directly test whether there is overlapping activation of hand representation and arithmetic processing. Using fMRI, the first study showed that simple arithmetic and finger discrimination tasks induce common activations in the horizontal IPS and posterior superior parietal lobule in adult participants (Andres et al., 2012). The relation was found to be stronger for subtraction compared to multiplication problems and for the left compared to the right hemisphere. A second study, focused on the neural networks involved in different numerical tasks (i.e., symbolic and non-symbolic number comparison, symbolic and non-symbolic addition and counting tasks) and their relationship to activations underlying finger representations (i.e., a guided finger movement task) and saccades in children (Krinzinger et al., 2011). The contribution of the finger-related network, including the ventral precentral sulcus, the supplementary motor area and the dorso-lateral prefrontal cortex, was stronger for the addition than for the comparison task.

Importantly, behavioral and neurofunctional data support the existence of operation-specific networks. Behaviorally, adults and children have been shown to rely more on retrieval strategies for multiplication problems compared to subtraction problems, which have been shown to require more on quantity manipulation (LeFevre et al., 1996; Fayol and Thevenot, 2012; Barrouillet and Thevenot, 2013). Indeed, language processes have been found to be more relevant for solving multiplication problems whereas visuo-spatial processes for solving subtraction tasks (Lee and Kang, 2002; Boets and De Smedt, 2010; De Smedt and Boets, 2010). Neurofunctionally, the brain networks involved in different operations have shown to be partially distinct (Fehr et al., 2007). Multiplication problems have been shown to elicit greater activation within the fronto-temporal network subtending verbal processing whereas subtraction problems have been found to elicit greater activations within the intraparietal sulcus, which is involved in numerical magnitude manipulation (Prado et al., 2011). Importantly, this dissociation has been found to increase developmentally indicating an increased differentiation in the network used to solve each operation (Prado et al., 2014). These results highlight how proficiency in single-digit problems may be achieved through different brain regions supporting different strategies.

Strategies used in school when learning how to solve arithmetic problems could shape the neural networks involved in processing multiplication and subtraction problems. Indeed, children are encouraged to retrieve multiplication problems by using a rote learning approach (Campbell and Xue, 2001) whereas subtraction problems are taught by means of procedures without emphasizing memorization (Dehaene, 1992; Campbell and Xue, 2001; Thevenot and Barrouillet, 2006; Barrouillet et al., 2008). Finger-based strategies are used predominantly to solve subtraction problems (Baroody, 1987; Siegler, 1987), and therefore, differential relation of finger-related activations are expected depending on operation type.

Although evidence suggests a close functional and representational link between fingers and arithmetic, no study has directly investigated how finger-related activation is related to skill in children, or whether these relations depend on operation type. Moreover, we do not know the specific contributions of finger representation (i.e., somatosensory activation) vs. finger movement (i.e., motor activation). Therefore, the aim of the present study was to understand skill-based effects in finger-related activation in both somatosensory and motor cortex during single-digit operations. We specifically used subtraction and multiplication problems because they have shown the greatest dissociation behaviorally and neurofunctionally (LeFevre et al., 1996; Prado et al., 2011, 2014; Fayol and Thevenot, 2012; Barrouillet and Thevenot, 2013). First, we tested whether different operations recruit these areas differently. Subtraction problems require greater quantity manipulation (Dehaene et al., 2003; Barrouillet et al., 2008) and multiplication problems rely more on verbal retrieval (Prado et al., 2011, 2014). In addition, finger movement selectively interfered with addition and subtraction problems but not with multiplication problems (Michaux et al., 2013). Therefore, we expected overall greater involvement of somatosensory and motor areas in subtraction problems. This would be consistent with the greater overlap between numerical processing and finger discrimination found for subtraction than multiplication problems in adults (Andres et al., 2012). Second, we tested the relationship between skill and amount of activation in finger related cortex. Studies on finger gnosia have shown that greater finger discrimination skill was predictive of future arithmetical skill (Noël, 2005; Gracia-Bafalluy and Noël, 2008). We therefore expected that children with better finger somatosensory representations would show higher accuracy. This may be associated with less activation because a recent study on passive sensory finger stimulation found decreased activity with increased spatial acuity of pinch grip in the somatosensory area (Ladda et al., 2014). Conversely, because children with math difficulties have been found to rely more on finger-based strategies (Alibali and DiRusso, 1999; Geary, 2004, 2005), we expected activation in finger motor areas to be related to lower accuracy.

## Materials and Methods

### Participants

Forty children (23 females) between 8 and 13 years of age were scanned. One participant was excluded for accuracy in the scanner beyond three SD from the average. Thus, 39 children were retained based on standardized testing performance and fMRI scan quality. All participants had a full-scale IQ standard score greater than 85 on the Wechsler Abbreviated Scale of Intelligence (WASI; Wechsler, 1999) with a group average of 116.3 [SD = 13.8, range (86–144); see Table 1]. To ensure participants had no mathematical difficulty, children had an 85 or above [mean = 106.3, SD = 12.2, range (88–143)] score on the Math Fluency (MF) subtest of the Woodcock–Johnson III (Woodcock et al., 2001). In this task, participants have 3 min to solve one-digit addition, subtraction, and multiplication problems. A timed task was chosen because it is an index of automaticity of procedural strategies and penalizes children that rely on lengthy and immature back-up strategies (Russell and Ginsburg, 1984; Fayol and Thevenot, 2012). Finally, all children performed 60% or higher on the fMRI tasks.

**Table 1:** **Age and standard scores for the 39 participants**.

	**Average (SD)**	**Max–Min**
Age (year:month)	11 (1:5)	8:2–13:4
WASI-IQ^*^	116 (13.5)	86–144
WJ-III-Math Fluency^*^	107 (12.4)	88–143

SD, standard deviation; WASI, Wechsler Abbreviated Scale of Intelligence; WJ-III, Woodcock–Johnson III. ^*^Standard Scores with average 100 (SD = 15).

Written consent was obtained from children and their legal guardians. The Institutional Review Board at Northwestern University approved all experimental procedures before data collection.

### fMRI Subtraction and Multiplication Tasks

Participants performed a subtraction and a multiplication task in the fMRI scanner and were required to judge if a proposed outcome was correct or incorrect responding with the right hand. For each task, based on previous research (Stazyk et al., 1982; Cooney et al., 1988; Siegler, 1988; Ashcraft, 1992; Campbell and Xue, 2001; De Brauwer et al., 2006), twelve small and 12 large one-digit problems were included. Problems involving 0 (e.g., 6 – 0 or 6 × 0) or 1 as one of the terms (e.g., 7 – 1 or 7 × 1), and ties (e.g., 4 – 4; 4 – 2) were excluded from the experiment but were used for practice purposes (i.e., 12 true and 12 false problems). For the subtraction task, problem size was determined by whether the difference between the terms was greater than three (e.g., 5 – 3 and 8 – 3 for small and large problems respectively). For the multiplication task, problem size depended on the size of the operands: small problems had the two operands equal or smaller than 5 (e.g., 2 × 4) and 12 large problems had both operands larger than 5 (e.g., 6 × 9). In both tasks, each problem was repeated twice with a true answer and once with a false answer, yielding 72 trials for each problem type (36 small problems and 36 large problems). For the subtraction task, false answers were generated by either adding 1 or 2 (e.g., 6 – 2 = 5) or subtracting 1 (e.g., 6 – 2 = 3) from the correct answer. For the multiplication task, the false answer was the correct result of the adjacent fact by adding or subtracting 1 to the first operand (e.g., 20 or 28 as the false answer to 6 × 4). Twenty-four null trials were included to control for motor responses for each task. In these trials participants had to respond when a blue fixation square turned red.

**FIGURE 1:**
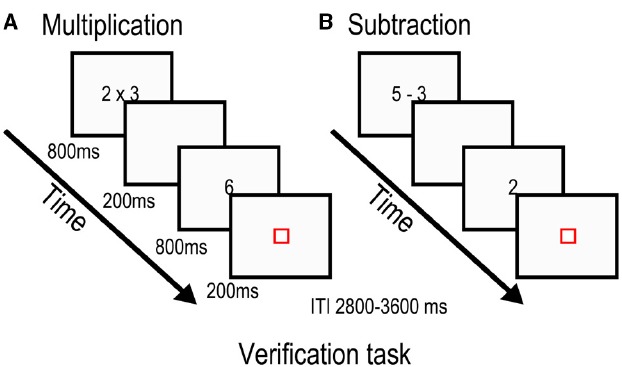
**Stimulus presentation for the multiplication task (A) and subtraction task (B).** Participants were asked to evaluate whether the proposed answer was the correct solution to the previously seen arithmetic problem.

Stimulus presentation was fixed and identical for the two tasks (Figure 1). The first stimulus was presented for 800 ms before being replaced by a blank screen for 200 ms. The second stimulus was also presented for 800 ms, but was followed by a red fixation square for 200 ms. The red square indicated the need to give a response during a variable interval ranging from 2800 to 3600 ms. Null trials were composed of a blue square that lasted for the same duration as the experimental conditions and participants had to press a button when it turned red. Finally, each run ended with 22 s of passive visual fixation. Each task was subdivided in two approximately 4.3 min blocks to allow for some resting time.

### Experimental Protocol

Participants were familiarized with the tasks and the fMRI environment during a practice session after giving informed consent and having completed standardized testing. During this session, they learned to minimize head movement in a mock fMRI scanner by means of an infrared-tracking feedback device and practiced all tasks. This session was completed within a week prior to actual fMRI data acquisition. In the fMRI scanner, each task was split into two 4-min runs. The timing and order of trials within each run were optimized for estimation efficiency using optseq2^1^. Behavioral responses were recorded using an MR-compatible keypad placed in the right hand. Stimuli were generated using E-prime software (Schneider et al., 2002) and projected onto a translucent screen that was viewed through a mirror attached to the head-coil.

### fMRI Data Acquisition

Images were collected using a Siemens 3T TIM Trio MRI scanner (Siemens Healthcare, Erlangen, Germany) at CTI, Northwestern University’s Center for Translational Imaging. The fMRI blood oxygenation level dependent (BOLD) signal was measured with a susceptibility weighted single-shot echo planar imaging (EPI) sequence. The following parameters were used: TE = 20 ms, flip angle = 80 s, matrix size = 128 × 120, field of view = 220 × 206.25 mm, slice thickness = 3 mm (0.48 mm gap), number of slices = 32, TR = 2000 ms. Before functional image acquisition, a high resolution T1 weighted 3D structural image was acquired for each subject (TR = 1570 ms, TE = 3.36 ms, matrix size = 256 × 256, field of view = 240 mm, slice thickness = 1 mm, number of slices = 160).

### fMRI Preprocessing

Data analysis was performed using SPM8^2^. After discarding the first six images of each run, functional images were corrected for slice acquisition delays, realigned to the first image of the first run and spatially smoothed with a Gaussian filter equal to twice the voxel size (4 mm × 4 mm × 8 mm full width and half maximum). Prior to normalizing images with SPM8, we used ArtRepair (Mazaika et al., 2007, 2009)^3^ to suppress residual fluctuations due to large head motion and to identify volumes with significant artifact and outliers relative to the global mean signal (i.e., 4% from the global mean). Volumes showing rapid scan-to-scan movements of greater than 1.5 mm were excluded via interpolation of the two nearest non-repaired volumes. Interpolated volumes were then partially deweighted when first-level models were calculated on the repaired images (Mazaika et al., 2007). Finally, functional volumes were co-registered with the segmented anatomical image and normalized to the standard T1 Montreal Neurological Institute (MNI) template volume (normalized voxel size, 2 mm × 2 mm × 4 mm). Scan quality was determined by the number of replacements in each functional run: up to 5% of replaced scans, but no more than four consecutive replacements, were accepted for each run.

### fMRI Processing

Event-related statistical analysis was performed according to the general linear model. Activation was modeled as epochs with onsets time-locked to the presentation of the first stimulus and with a duration matched to the length of the trial (i.e., 2 s). Trials were classified for problem type (true, false) and for problem size (small, large). However, only true trials were considered of interest in behavioral and fMRI analyses because for false trials it is impossible to determine if the answer was rejected by using a calculation procedure or relying on alternative strategies such as parity judgment or estimation (Lemaire and Reder, 1999). Moreover, during false trials, conflict detection and error monitoring processes could affect activation patterns (van Veen and Carter, 2002, 2006; Ferdinand and Kray, 2014; Ullsperger et al., 2014). Null trials were further modeled in a separate regressor. All epochs were convolved with a canonical hemodynamic response function. The time series data were high-pass filtered (1/128 Hz), and serial correlations were corrected using an autoregressive AR (1) model.

### Motor and Somatosensory Region of Interest Definition

**FIGURE 2:**
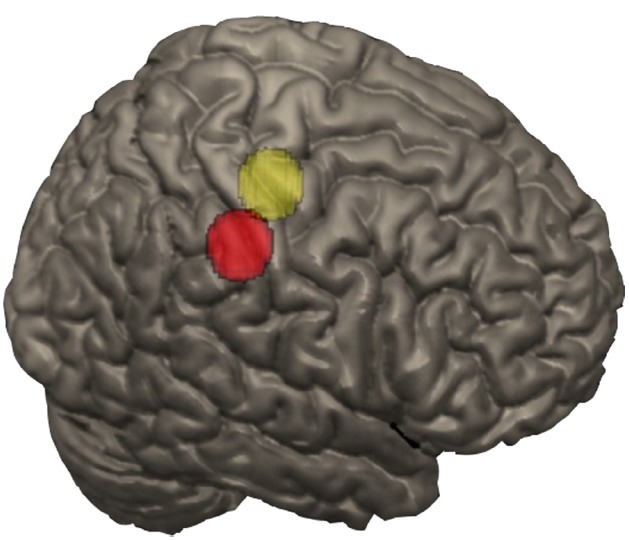
**Nine millimeter radius spheres for the Motor (yellow) and Somatosensory (red) ROIs**.

To isolate finger-related activation, finger somatosensory and motor areas were defined using Neurosynth, a large-scale automated synthesis of human functional neuroimaging data^4^. Spheres of 9 mm (i.e., 196 voxels) were created by identifying the peaks in the pre- and postcentral gyrus forward inference maps for fingers as centers. Only the right hemisphere was considered since participants were using their right hand to give their response and the left hand was available as support for calculations. The somatosensory peak was at 46, –30, 44 and the motor peak was at 38, –22, 60 in MNI coordinates (Figure 2).

### fMRI Analyses

Voxel-wise regressions were run within the two region of interests (ROIs) to investigate the relation with accuracy. Age was entered as control variable and accuracy for each task or condition as variable of interest. An uncorrected height threshold was set at *p* < 0.01 for the contrast maps and Monte Carlo simulations (3dClustSim) were used to set the extent threshold at *p* < 0.05 corrected. Statistical results are reported in the MNI coordinate space.

## Results

### Behavioral

Repeated measures ANOVAs were run with operation and problem size as within subject variables on accuracy and reaction times (RTs). Large problems were significantly slower [*F*_(1,38)_ = 78, *p* < 0.001] and less accurate [*F*_(1,38)_ = 51, *p* < 0.001] than small problems (Table 2). Moreover, for accuracies only, the interaction indicated that the problem size effect was larger for multiplication problems [*F*_(1,38)_ = 19, *p* < 0.001]. All other effects were non-significant.

**Table 2:** **Accuracy and reaction times (RTs) for the subtraction and multiplication tasks**.

**Task**	**Accuracy (SD)**	**RT (SD)**
Subtraction	91 (8.9)	1173 (336)
Small problems	93.7 (6.6)	1058 (318)
Large problems	88.3 (12.4)	1252 (380)
		
Multiplication	88 (10.5)	1120 (358)
Small problems	96.7 (5.2)	960 (329)
Large problems	79 (18.4)	1239 (380)

SD, standard deviation.

Age was significantly correlated with subtraction accuracy (*r* = 0.403, *p* = 0.011) and only marginally to multiplication accuracy (*r* = 0.309, *p* = 0.056), but not with RTs. Controlling for age, accuracies to the two tasks were also correlated (*r* = 0.428, *p* = 0.007).

### Operation Related Activation

**FIGURE 3:**
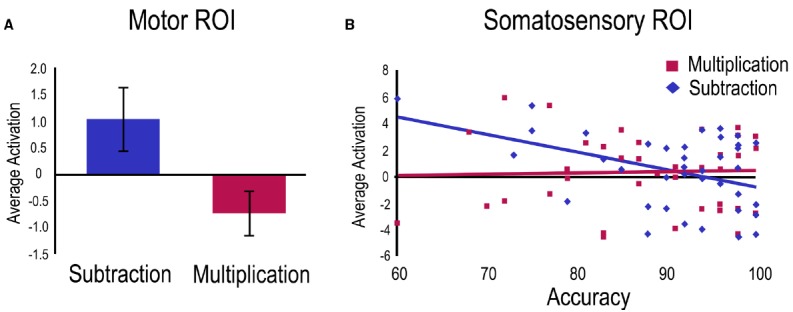
**Operation related activation for the subtraction vs. multiplication contrast.** For visualization purposes, betas from significant clusters have been extracted for **(A)** the main effect in the Motor ROI showing greater overall activation for the subtraction task, and **(B)** the activation related to accuracy in the Somatosensory ROI only for the subtraction task.

A first regression was run on the subtraction vs. multiplication contrast with age as control variable and accuracy as a predictor. A main effect of task was found within the Motor ROI indicating greater activation for subtraction problems compared to multiplication problems (Figure 3A). Within the Somatosensory ROI, a negative relation was significant with subtraction accuracy (Figure 3B).

### Multiplication Task

**FIGURE 4:**
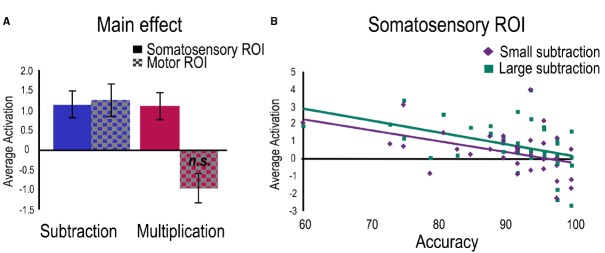
**Activation related to the two tasks separately.** For visualization purposes, average betas have been extracted for **(A)** the clusters showing main effects in both ROIs for each task, and **(B)** the significant clusters showing a negative relation with accuracy for each problem size only found in the subtraction task.

Separate regressions were then run for each task with age as control variable and accuracy as a predictor. For the multiplication task, a positive main effect was found within the Somatosensory ROI and a marginally negative main effect in the Motor ROI (Figure 4A). No relation with accuracy was found significant. To investigate problem size, a regression for the large vs. small contrast was run with age as control variable and accuracies to small and large problems as predictors. No main effect or relations with accuracies were found significant.

### Subtraction Task

For the subtraction task, significant positive main effects were found in both ROIs (Figure 4A) and the negative relation between accuracy and activation was confirmed within the Somatosensory ROI. A regression to investigate problem size (large vs. small contrast) was therefore run with age as control variable and accuracies to small and large subtraction problems as predictors. A main effect was found in the Somatosensory ROI indicating greater engagement for large problems compared to small problems. Separate regressions were also run for large and small problems separately. For large problems, positive main effects were found in both ROIs and a significant negative relation was found with accuracy only in the Somatosensory ROI. For small problems, only the negative relation with accuracy was significant (Figure 4B).

## Conclusion

Evidence suggests that finger representation and finger-based strategies play an important role in learning and understanding arithmetic (Alibali and DiRusso, 1999; Geary, 2005; Gracia-Bafalluy and Noël, 2008; Newman and Soylu, 2013). However, no study has investigated in children the neural underpinnings of finger representation and finger movement involved in arithmetic and their relation to skill. This is the first study to specifically investigate skill based effects in finger-related areas in somatosensory and motor cortex during single-digit problems. Previous studies have shown that different operations rely on different processes (Arsalidou and Taylor, 2011; Ashkenazi et al., 2013) and that interference from finger movements depends by operation type (Michaux et al., 2013); therefore we also tested whether hand-related activation varied depending on the operation. We compared activation for subtraction and multiplication problems in independently localized finger somatosensory and motor areas and tested whether activation was related to skill.

Comparing activations for the two operations, we found that children recruited motor areas more for subtraction problems suggesting that they were more likely to support their mental processes with finger-based back up strategies. This is consistent with evidence suggesting that subtraction problems require more quantity manipulation (Dehaene et al., 2003; Barrouillet et al., 2008) and multiplication problems rely more on verbal retrieval (Prado et al., 2011, 2014). Against our initial hypothesis, we did not find finger motor activation to be related to skill. Because previous behavioral studies have found that children with math difficulty rely more on finger-based strategies than typically performing peers (Alibali and DiRusso, 1999; Geary, 2004, 2005), we expected to find a relation between motor activation and skill. Additionally, we showed no difference in motor activation with problem size. A possible explanation is that children were relying on some finger-based strategy irrespective of problem size but the use of this strategy did not yield better performance. Conversely, because children were discouraged to move in the scanner, it could be that they were inhibiting explicit finger movements but still showed activation in the motor area. Consistent with the latter explanation is the study from Tschentscher et al. (2012). The authors report activation in the motor and premotor areas contralateral to the preferred hand for counting when perceiving numbers, digits or written words, despite the absence of overt hand movement. Finger-based and counting strategies may become internalized to the point of influencing adult performance during numerical tasks (Di Luca et al., 2006; Di Luca and Pesenti, 2008; Klein et al., 2011). Our result suggests that the observed influence might be the consequence of an implicit activation of the areas supporting these finger-based strategies.

In both tasks, we found significant activation in the somatosensory area suggesting an implicit activation of finger representation. Indeed, several studies have shown that finger representation is automatically recruited when processing numerical information (Di Luca et al., 2006; Di Luca and Pesenti, 2008; Badets and Pesenti, 2010; Badets et al., 2010). A critical finding of our study is that activation in the somatosensory area was related to performance only for subtraction problems. Studies have shown that finger gnosia, that is the quality of an individual’s finger representation, in children was related to arithmetical skill and training finger representation also improved performance to arithmetic tasks (Fayol et al., 1998; Noël, 2005; Gracia-Bafalluy and Noël, 2008; Reeve and Humberstone, 2011). Our result suggests that finger representation has greater functional involvement for operations requiring greater quantity manipulation. Importantly, the relation indicated that children with low performance engaged somatosensory areas more than children with higher performance. A first possible explanation is that low performers relied more on an immature finger-based strategy whereas high performers possibly retrieved the answer. However, this explanation seems unlikely because it should also reflect in a negative relation within the motor area, but this was not found. The second and more likely explanation is that activation in the somatosensory areas is negatively related with the quality of fingers representation: participants with greater finger gnosia have lower levels of activation within the somatosensory area. In support, studies on sensory finger stimulation showed decreased activity within the somatosensory area after training along with increased spatial acuity (Ladda et al., 2014). Therefore, the negative relation may indicate that children that performed better on subtraction problems were also those with finer finger representation.

We also found that the engagement of the somatosensory cortex was modulated by problem size only within subtraction problems. Large problems engaged the somatosensory area more than small problems. We suggest that more fingers are needed to represent large numbers thus requiring greater engagement of the somatosensory area. It can be argued that large problems might be harder and induce more finger-based backup strategies. However, these problems did not engage more motor activation compared to small problems suggesting that children were not more likely to rely on finger-based strategies. This is in contrast to previous studies that have shown greater activation in motor related areas for arithmetic tasks. In a study using single-digit multiplication problems, along with activity found in retrieval areas, the authors also found increased activity for harder problems in premotor and frontal cortices (Jost et al., 2009). A network including the IPS, the inferior frontal gyrus and the precentral gyrus also showed increased activation for larger single-digit addition problems (Stanescu-Cosson et al., 2000). It can be hypothesized that larger problems required greater computation thus involving more motor hand support. The differences between our study and previous ones could be ascribed to task presentation differences. In Jost et al. (2009) participants had to produce the answer to consecutive multiplication and addition problems. This paradigm might have increased working memory load thus increased the reliance on finger-based strategies. In Stanescu-Cosson et al. (2000) study, additions problems were presented in blocks divided by problem size. This presentation format might have induced different strategies depending on problem size due to differences in cognitive load between the blocks. Because large problems are also usually harder, participants might have more often used back-up strategies for such blocks. In our study, children had to judge the accuracy of small and large intermixed problems. This presentation might have decreased the chances of using different solutions depending on problem size.

Two alternative explanations for these results need to be discussed. First, studies have shown that the visual presentation of symbols activates a complex network including sensorimotor, premotor and motor areas corresponding to the graphic movements involved in tracing or writing (Longcamp et al., 2003, 2005; James and Gauthier, 2006). However, because the stimuli presented were single-digits in both tasks, it is unlikely that the differences in motor and sensorimotor activations found in our study can be ascribed to differences in the network subtending such movements. It is unlikely that only the symbols for the subtraction task elicited a graphic motor network. Our results also show a relation between performance and activation in the somatosensory area, which is not consistent with graphic-related motor activations. Additionally, Exner’s area, known as the “graphic motor image center” in the frontal lobe, is situated more anteriorly compared to our ROIs (Roux et al., 2009; Purcell et al., 2011, for a meta analysis). Second, a limitation of this study is the use of a manual response. Indeed, studies have shown that motor responses induce activations both in the contralateral and ipsilateral hemisphere, which could interact with our results (Buetefisch et al., 2014). However, previous studies also show that activation found in the ipsilateral hemisphere is spatially distinct from activation found during the contralateral task (Cramer et al., 1999). If contralateral activation is modulated by task difficulty, where greater cognitive load increases ipsilateral motor activation (Buetefisch et al., 2014), our brain-behavior results should have shown a relation with multiplication problems rather than subtraction problems because they show a larger problem size effect. Additionally, because the two tasks were well matched in overall accuracies, it is unlikely that the differences observed between tasks were induced by greater ipsilateral activation for correct responses only in the subtraction task. Finally, the amount of ipsilateral motor button response cannot explain differences in activation because in both tasks participants required a response for each trial (i.e., true or false).

Overall, our results support the importance of fine-grained finger representation (i.e., finger gnosia) in performing subtraction problems and are the first evidence for a functional role of the somatosensory finger area in proficient arithmetical problem solving, in particular for those problems requiring quantity manipulation. Although training studies show a causal role of finger gnosia in arithmetical skill (Gracia-Bafalluy and Noël, 2008), future studies should investigate the differential impact depending on operation. Our results suggest that training should have more pronounced effects on subtraction compared to multiplication. Studies should also investigate the neurofunctional changes associated with finger training and their relation to performance. It is possible that training may enhance the relation between amount of activation and accuracy in somatosensory cortex, or alternatively, it could weaken the relationship due to greater benefit for low skill participants. Finally, because children with difficulties in learning mathematics show lower finger gnosia (Strauss and Werner, 1938; Noël, 2005; Costa et al., 2011), it would be interesting to investigate whether these children differ in their ability to recruit somatosensory areas during arithmetical problem solving and whether it relates to performance. A logical extension of our results would predict that children with mathematical learning disability would show even greater recruitment of somatosensory cortex during arithmetic problems.

Currently, educational practices have considered finger counting as a tool to introduce the transformations associated to each operation and as an initial support to alleviate working memory (Alibali and DiRusso, 1999; Geary, 2005). However, little attention has been given to the types of counting strategies and their implications on performance. Great cultural variability has been described and different types of finger counting strategies possess different properties (Bender and Beller, 2012). It is therefore important to understand developmentally if different properties such as dimensionality, base and sub-base values, regularity or number of distinct finger configurations might prove more efficient in fostering understanding of numerical operations. For example, some studies suggest that the spatial-numerical association, which in turn influences arithmetical processing (Knops et al., 2009, 2014), might be influenced by finger counting strategies. Given our results showing motor and somatosensory activations dependent on operation type, systematically studying the influence of different finger strategies might be a promising area of research with direct educational implications.

In conclusion, these results support educational practices encouraging the use of fingers as functional link between numerical quantities and their symbolic representation as well as an external support for learning arithmetic problems. These results also encourage educational practices to focus on finger discrimination as a precursor of numerical and arithmetical skill. Finally, although the National Mathematics Advisory Panel (2008) suggests that children should achieve automatic retrieval for all arithmetic facts, regardless of the operation, our study strengthens the hypothesis that different arithmetic operations are processed in distinct ways and successful performance might be achieved through at least partially different neurofunctional processes.

## Author Contributions

IB collected, analyzed and interpreted the data, conceived the work and wrote the manuscript; JRB conceived the work, interpreted the data, and critically revised the work.

### Conflict of Interest Statement

The authors declare that the research was conducted in the absence of any commercial or financial relationships that could be construed as a potential conflict of interest.

## Footnotes

1 http://surfer.nmr.mgh.harvard.edu/optseq/

2 www.fil.ion.ucl.ac.uk/spm

3 http://cibsr.stanford.edu/tools/human-brain-project/artrepair-software.html

4 www.neurosynth.org
